# Downward Vertical Gaze Palsy As A Prominent Manifestation Of Episodic Ataxia Type 2: A Case Report

**Published:** 2013

**Authors:** Reza SHERVIN BADV, Ali NIKSIRAT

**Affiliations:** 1 Pediatric Neurologist, Department of Pediatrics, Zanjan University of Medical Sciences, Zanjan, Iran; 2 Legal Medicine Research Center, Legal Medicine Organization, Tehran, Iran

**Keywords:** Episodic ataxia type 2, Downward vertical gaze palsy, Acetazolamide, Normal brain function

## Abstract

**Objective:**

Episodic ataxia type 2 (EA2) is an inherited autosomal dominant disorder characterized by intermittent ataxia, nausea, vomiting, dysarthria, or nystagmus.

We report a case of EA2, which downward gaze palsy exists as a common sign in all her attacks. Responsiveness of EA2 to acetazolamide was observed in this patient.

## Introduction

Intermittent and recurrent ataxias are described in several vestibular, metabolic, vascular, and genetic disorders (1). Episodic ataxias (EAs) are characterized by recurrent episodes of cerebellar ataxia, vertigo, dysarthria, and nystagmus, starting in childhood and lasting for minutes or hours, with otherwise normal brain functions (2). Data is not available on the exact incidence of EAs, but it seems to occur in 3 to 5 people per 100,000 in the population (3). Nowadays there are seven recognized EA syndromes (numbered 1 to 7). These disorders share some common features: all are channelopathies with autosomal-dominant inheritance pattern and are responsive to acetazolamide (2). EA1 and EA2 are clinically well described, while the others are exceptionally rare (3). Both EA1 and EA2 are the result of ion channel mutations. Mutations in a potassium channel gene (KCNA1) located on chromosome 12p13 underlie EA1, and in a voltage-dependent calcium channel gene (CACNA1A) located on chromosome 19p13 can lead to EA2 (4). EA1 is characterized by brief episodes of ataxia lasting seconds to minutes, with onset in early childhood and often induced by startle or exercise, emotional stress, sudden change in posture, and by the continuous interictal myokymia (5,6). EA2, spinocerebellar ataxia type 6 (SCA6), and familial hemiplegic migraine all represent allelic mutations in the same calcium channel gene on chromosome 19p13 (7). EA2 is the most common of all episodic ataxias. The attacks are often associated with nausea, vomiting, dysarthria, or nystagmus, and last several hours to days. The frequency of these paroxysmal events varies from daily to once a year. The attacks are triggered by physical exertion, stress, or nonspecific febrile illness. In contrast with EA1, the attacks are not related to sudden movement. Approximately 50-70% of all patients with EA2 are responsive to treatment with acetazolamide, concerning both the frequency and the severity of the attacks (8). Sodium valproate may be a necessary adjunct to acetazolamide (9).

## Case presentation

A 6-year-old girl was presented with a 3 year-long history of episodic instability, upward gaze, vomiting, vertigo, and cerebellar signs (dysmetria and dysarthric speech). She was developmentally normal. Her parents were not consanguine. Her family history showed no cases of similar illness. The first attack had begun at 21 months of age with sudden onset of upward gaze and nausea after a febrile disease, which led to hospitalization with suspicious diagnosis of seizure and lasted for two days. Thereafter, her mother mentioned the recurrence of similar attacks after every febrile disease, which did not respond to usual symptomatic treatments. 

Downward gaze palsy existed as a common sign in all her attacks. The attacks occured approximately once a month and between them, she was completely normal. Her symptoms were relieved during sleep, and were not related to bath, foods and exercise. Blood cell counts, urinalysis, blood and urine amino acid chromatography, liver and renal function tests, blood gases, serum biochemistry and electrolytes all were in normal ranges. Auditory-evoked potentials, cerebrospinal fluid (CSF) studies, electroencephalogram (EEG), and brain magnetic resonance imaging (MRI) were also normal. The patient was hospitalized several times with various diagnoses, such as inherited metabolic disorder, basilar migraine, and epilepsy, and was treated with several drugs that were not effective. Then, suspicious diagnosis of EA2 was made and acetazolamide was prescribed. The frequency and severity of her symptoms decreased after receiving acetazolamide. Approximately 2 months after the last treatment (acetazolamide 250 mg twice a day orally), the patient’s attacks were completely resolved. At present she is a 7-year old excellent student and goes to school regularly.

## Notes

**In conclusion, **EA2 is an autosomal dominant disorder characterized by intermittent vertigo, ataxia, and interictal gaze-evoked nystagmus (10). The underlying cause of EA2 is a genetic alteration in the voltagedependent calcium channel (CACNA1A) (11). Episodes may occur spontaneously or may be triggered by stress and exertion (5,12). In our patient, episodic attacks was always occurring after a period of fever and was not related to bath, carbohydrate-rich foods, drinks, tea, caffeinated food, and exercise. Episodic ataxia most typically presents itself in the teenage years, but the time of onset can range from early childhood to early adulthood. However, EA2 does not shorten life span but significantly influences the quality of life. The clinical features and the family history are the basis for diagnosis.

Molecular diagnosis is available on a research basis. MRI may show selective atrophy of the cerebellar vermis (7).

Our patient’s family history and all of her workup were negative, so, it seems that a high index of suspicion is necessary. After treatment with acetazolamide, her attacks were controlled. Acetazolamide, a carbonic anhydrase inhibitor, is effective in reducing the frequency of ataxic episodes in some patients (8,13).

The drug’s mechanism of action is alteration of intracellular pH. The attacks are precipitated by high intracellular pH values, exercise, and stress through hyperventilation and consequent alkalosis (11). Acetazolamide decreases the intracellular pH, which reduces potassium conductance and thereby restores excitability and resting activity of the neurons (14). Nystagmus with features of rebound nystagmus is seen in between attacks (15), but interictally, our patient was completely normal. Although patients are asymptomatic between attacks, neurologic examination may reveal ocular abnormalities, including downbeating or gazeevoked nystagmus, abnormal optokinetic nystagmus, hypermetric saccades, saccadic pursuit, or myokymia (16,17).

Our patient was hospitalized several times with incorrect diagnosis and she had no improvement. Considering that our diagnosis was made based on the manifestations of EA2, her attacks were completely controlled and resolved after treatment with acetazolamide. We did not perform mutation analysis, but the diagnosis of EA2 was confirmed based on the clinical manifestation and responsiveness to acetazolamide, but not to other drugs. Singhvi et al. also reported a case of EA2 that was confirmed according to clinical manifestation and responsiveness to acetazolamide (18). It is obvious that mutation analysis is very helpful, but it is not obligatory and patients can be diagnosed by experimental prescription and spending less money.

Downward vertical gaze palsy as a prominent ictal sign has not yet been reported in any literature. Presence of downward vertical gaze palsy in the attacks of our patient shows that in similar cases of episodic ataxias accompanied by ictal sign, diagnosis of EA2 should be considered.
